# Supplementary material for: Mechanisms governing the pioneering and redistribution capabilities of the non-classical pioneer PU.1
Source: Nat Commun. 2020 Jan 21;11:402. doi: 10.1038/s41467-019-13960-2 (PMC6972792; doi:10.1038/s41467-019-13960-2)
Supplement: Supplementary file 7 — Source data [file 41467_2019_13960_MOESM7_ESM.zip › Source_Data/Figure6/Figure6B_MotifScanOutput/ETS1_PU.1/homerResults/motif6.similar.html]

motif6

## Information for motif6

A
G
T
C
C
G
T
A
A
C
G
T
A
G
T
C
A
G
T
C
A
C
T
G
A
T
C
G
  
Reverse Opposite:  

A
T
G
C
A
G
T
C
A
C
T
G
A
C
T
G
C
G
T
A
C
G
A
T
C
T
A
G
  

|  |  |
| --- | --- |
| p-value: | 1e-395 |
| log p-value: | -9.096e+02 |
| Information Content per bp: | 1.908 |
| Number of Target Sequences with motif | 3315.0 |
| Percentage of Target Sequences with motif | 20.94% |
| Number of Background Sequences with motif | 2307.1 |
| Percentage of Background Sequences with motif | 7.19% |
| Average Position of motif in Targets | 98.1 +/- 48.6bp |
| Average Position of motif in Background | 101.2 +/- 68.4bp |
| Strand Bias (log2 ratio + to - strand density) | 0.0 |
| Multiplicity (# of sites on avg that occur together) | 1.09 |
| Motif File: | file (matrix) reverse opposite |

### Similar de novo motifs found

|  |  |  |  |  |  |  |  |
| --- | --- | --- | --- | --- | --- | --- | --- |
| Rank | Match Score | Redundant Motif | P-value | log P-value | % of Targets | % of Background | Motif file |
| 1 | 0.896 | C T G A A G T C C T G A A C G T A G T C A G T C A C T G A T C G A G T C A T C G A G C T | 1e-292 | -673.839919 | 9.53% | 1.92% | motif file (matrix) |
| 2 | 0.852 | T C G A T C G A C T G A T A G C T G A C C T A G A T C G T G C A A G C T T C A G | 1e-133 | -307.154062 | 7.71% | 2.66% | motif file (matrix) |
| 3 | 0.761 | T A C G T A G C T A G C C T A G T A C G T C G A C T G A T C A G G T A C T C A G T A C G T G A C A T C G T A C G | 1e-122 | -282.736410 | 10.61% | 4.71% | motif file (matrix) |
